# Research Progress on High-Protein Peanut (*Arachis hypogaea* L.) Varieties in China

**DOI:** 10.3390/plants14182917

**Published:** 2025-09-19

**Authors:** Zhuo Li, Yaru Zhang, Yinghui Liu, Yi Fan, Ding Qiu, Zhongfeng Li, Fangping Gong, Dongmei Yin

**Affiliations:** College of Agronomy, Henan Agricultural University, Zhengzhou 450046, China; lz20010318@163.com (Z.L.); zhangyaru24@163.com (Y.Z.); liuyinghui2023@163.com (Y.L.); 15537151951@163.com (Y.F.); qiuding1989@outlook.com (D.Q.); 13810414626@163.com (Z.L.)

**Keywords:** peanut, high-protein, breeding, traits, genealogy

## Abstract

Peanut (*Arachis hypogaea* L.) protein, as a precursor to various amino acids and bioactive peptides, determines the flavor and nutritional quality of peanut products. Therefore, high protein content is one of the target traits in advanced peanut breeding programs. In this review, we summarized the characteristics of all currently available high-protein peanut varieties in China and provided a comprehensive analysis of the genetic, physical characteristics, and disease resistance. These varieties mostly were developed through interspecific hybridization or selected from mutants of self-pollinated parents, primarily using the cultivars “Silihong” and “Baisha 1016” as main parental lines. In terms of disease resistance, although most high-protein peanut varieties can resist two to four types of disease, few varieties exhibit resistance to multiple diseases, and some varieties show no resistance for tested disease or lack sufficient experimental validation. The genetic basis of high-protein peanuts is relatively narrow, relying mainly on a small number of parental varieties. The findings of this review provide important references for high-protein peanut breeding, highlighting the existing problems and challenges in current breeding efforts and emphasizing the importance of broadening the genetic base, enhancing disease resistance breeding, and optimizing overall quality. This review offers theoretical and practical guidance for future breeding of high-quality, high-yield, and high-protein peanut varieties, contributing to the sustainable development and quality improvement of the peanut industry.

## 1. Introduction

Peanuts (*Arachis hypogaea* L.) are a globally significant legume crop, famous for their rich nutritional composition, which includes substantial amounts of protein, healthy fats, vitamins, and minerals [1,2,3]. As a versatile food source, peanuts play a critical role in human diets and various industrial applications. Conventional peanut varieties typically contain about 25–28% protein by weight. High-protein peanut varieties further enhance these nutritional benefits, offering an exceptional dietary option, especially in regions where animal protein is inaccessible or expensive [4]. In developing countries, where protein-energy malnutrition remains a pressing public health issue, these varieties can provide an affordable and sustainable source of nutrition. Moreover, their plant-based nature aligns with the dietary preferences of vegetarians, vegans, and individuals seeking to reduce meat consumption, serving as a complete protein source when complemented with other plant foods [5]. The market potential for high-protein peanuts is expanding rapidly, driven by health-conscious consumption trends and functional food innovations. In China, the world’s largest producer and consumer of peanuts, government initiatives aimed at improving nutritional security and reducing dependence on soybean imports are further stimulating demand for high-protein varieties. However, breeding such varieties poses considerable challenges, including the need to balance protein content with yield stability, oil quality, and resistance to biotic and abiotic stresses.

Peanut protein, as a precursor to various amino acids and bioactive peptides, determines the flavor and nutritional quality of peanut products [6]. Historically, peanut breeding programs in China have prioritized increasing yield, oil content, and disease resistance. Peanut breeding programs in China are undertaken by institutions and entities at various levels, including national-level research institutions, provincial academies of agricultural sciences, higher education institutions, municipal-level agricultural research institutes, and seed enterprises. According to data provided by the National Natural Science Foundation of China (NSFC) (https://kd.nsfc.cn/finalProjectInit, accessed on 1 August 2025), about 120 peanut-related NSFC projects were completed between 2020 and 2023. The primary objectives and directions of these projects mainly focus on the following aspects: high yield, high oil content, high oleic acid, high protein, disease resistance (e.g., bacterial wilt and leaf spot), stress tolerance (e.g., drought tolerance and resilience in poor soil conditions), suitability for mechanized harvesting (e.g., compact plant architecture and strong pegs), as well as the development of specialty varieties (e.g., for fresh consumption and roasting). Breeding peanut varieties with high seed protein content has become a promising method to overcome malnutrition and improve the nutritional and culinary value of peanut-based foods [7]. On a dry weight basis, crude protein content in peanuts ranges from approximately 24% to 36%, making it the second highest among oilseed crops after soybeans (36% to 51%) [8]. Moreover, peanuts contain fewer anti-nutritional factors compared to soybeans. Their favorable nutritional profile, high yield, and low cost have spurred growing interest in the use of peanut protein and its by-products in the food industry. Currently, high-protein peanuts are generally defined as peanuts with a protein content exceeding 28%. Protein content in peanut seeds is a quantitative trait controlled by multiple genetic loci and is often negatively correlated with seed oil content [7,9,10,11,12], which presents both opportunities and challenges for breeding high-protein peanut varieties. With the development of genomics technologies, such as Quantitative Trait Loci (QTL) mapping [7], Genome-Wide Association Studies (GWAS) [12,13], and gene editing, crop breeders can more precisely identify and utilize key genes controlling protein content. These tools are accelerating the development of improved high-protein peanut varieties. In peanut breeding, the negative correlation between seed protein content and oil content is consistent with the competitive metabolism and accumulation of oil and protein as the main storage compounds in peanut seeds [14]. Despite these advances, research dedicated to enhancing peanut seed protein content remains limited, particularly in the development of protein-associated molecular markers and the identification of functional genes regulating protein and amino acid metabolism. Furthermore, the frequently observed negative correlation between high protein content and elevated oleic acid or oil content complicates simultaneous trait improvement, necessitating strategic multi-gene pyramiding to optimize these competing traits. The genetic redundancy inherent in the allotetraploid peanut genome further impedes the identification of key regulatory genes, highlighting the critical need to leverage multi-omics approaches for elucidating the complex regulatory networks underlying seed protein accumulation.

Research on protein-related traits constitutes an important area of focus within these efforts. Here, we collected all the currently available data on high-protein peanut varieties (168) of China from the following sources: the Variety Registration Inquiry of the Seed Management Division of the Ministry of Agriculture and Rural Affairs (http://www.zys.moa.gov.cn/, accessed on 1 January 2025), the Peanut Database (http://peanut.cropdb.cn/variety/, accessed on 1 January 2025), the First Seed Industry Network (http://www.a-seed.cn/, accessed on 1 January 2025), the China Seed Industry Big Data Platform (http://202.127.42.47:6010/index.aspx, accessed on 1 January 2025), and related literature. At the same time, related information for their agronomic traits, quality traits, disease resistance, and suitable cultivation areas was summarized (Table S1). This review seeks to consolidate and analyze existing data on high-protein peanut varieties developed in China, providing a detailed assessment of their traits, performance, and the methodologies employed in their creation. By synthesizing this information, the paper aims to offer a clear picture of the current state of high-protein peanut breeding and to guide future research and development efforts.

## 2. Main Text

### 2.1. Suitable Regions for High-Protein Peanut Varieties in China

**Figure 1:**
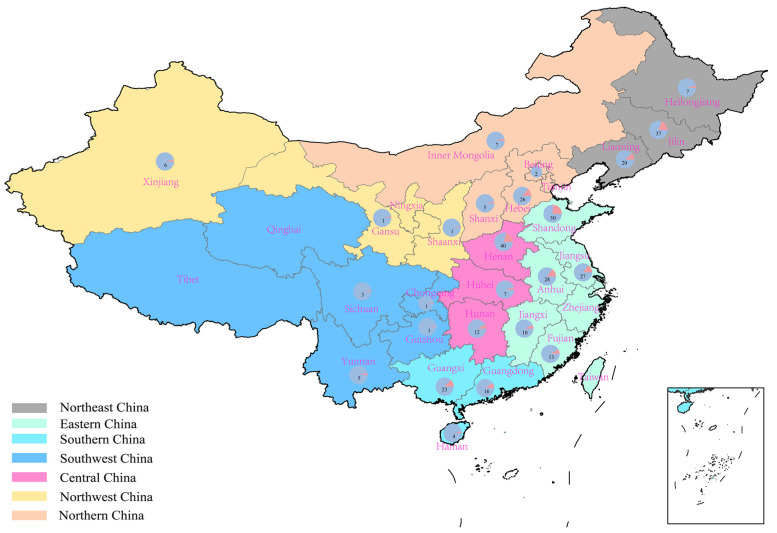
Distribution of suitable planting regions for high-protein peanuts in China [GS(2024)0650].

Peanuts are cultivated across multiple regions in China, including Northern China, Northeast China, Eastern China, Southern China, Southwest China, Central China and Northwest China [15]. These regions differ considerably in geographical and climatic conditions, agricultural practices, natural ecological features, economic development levels, and yield distribution. Among the 168 high-protein peanut varieties, the highest number of suitable varieties was identified in Eastern China, followed by Northeast China, Central China, Southern China, Northern China, Southwest China, and Northwest China. Specifically, Eastern China accounted for 128 high-protein peanut varieties, with Shandong representing the largest proportion (39%). Northeast China contained 64 high-protein peanut varieties, with Jilin contributed 58%. Central China had 59 high-protein peanut varieties, with Henan comprising 68% (Figure 1). High-protein peanut varieties are notably concentrated in Jilin, Henan, and Shandong. In contrast, the Southwest and Northwest regions show relatively fewer varieties, with Yunnan, Sichuan, and Xinjiang having comparatively higher counts within their respective areas. At the provincial level, Shandong had the highest proportion of suitable varieties at 38%, followed by Henan and Jilin at 25% and 24%, respectively. High-protein peanut varieties have been developed in all provinces except Ningxia, Qinghai, Taiwan, Tianjin, Tibet, and Zhejiang. The disparities in variety distribution among provinces are influenced by factors such as natural soil suitability, water availability, soil fertility, temperature conditions, light conditions, and accumulated temperature differences. Given the variations in climatic conditions and soil properties across different regions, the adoption of scientific crop rotation patterns in peanut cultivation practices can effectively enhance peanut yields, while improving soil nutrient status and optimizing the diversity of soil microbial communities. This practical approach can synergize with regional environmental adaptability and variety selection, thereby providing technical support for the stable growth of high-protein peanuts in different production areas. It is worth noting that the number of suitable varieties in a region does not necessarily reflect its inherent agronomic suitability or potential for high-protein peanut cultivation in those areas. Regional environmental conditions and cultivation practices significantly influence variety adaptability and performance. Moreover, different production regions exhibit distinct characteristics, which may affect the development and suitability of specific variety types. Future research should consider employing standardized growing conditions or statistical methods to account for environmental biases. Additionally, breeding efforts and technology promotion should be strengthened in regions with currently lower productivity to enhance the overall production and accessibility of high-protein peanuts.

### 2.2. Agronomic, Quality, and Yield Traits of High-Protein Peanut Varieties

Peanuts are rich in nutrients, including fats, proteins, flavonoids, anthocyanins, and other bioactive compounds. Among the 168 high-protein peanut germplasms in China, their protein content was above 28%. Specifically, 118 varieties (70.2%) had protein content between 28% and 29.9%, and 50 varieties (29.8%) had protein content above 30%. The average protein content of high-protein peanut varieties was 29.52%, ranging from 27.99% to 36.40%, with a coefficient of variation of 4.86%. This indicates that the protein content of most high-protein peanut varieties in China is below 30%, but the overall level of targeted breeding for high-protein peanuts is gradually improving. It is worth noting that high-protein peanuts still represent a small proportion of overall production; however, the number of such varieties has been increasing in recent years, reflecting growing emphasis on this trait. In addition, the average oil content was 50.90%, with a range of 36.40% to 59.32%, and a coefficient of variation of 5.75%. The average oleic acid content was 47.02%, with a range of 33.00% to 90.60%, and a coefficient of variation of 22.63% (Table 1). The considerable variation in protein and oleic acid content among varieties and across environments provides valuable insights for peanut cultivation and variety selection. Suitable varieties should be chosen according to local environmental conditions to optimize both nutritional quality and yield. The average growth period was 122.25 days, with a range of 100 to 138 days and a coefficient of variation of 7.00, indicating a relatively small fluctuation in growth period. Most of the high-protein peanut varieties collected were early-maturing varieties, with growth periods ranging from 100 to 130 days. Only 15 high-protein peanut varieties had growth periods exceeding 130 days, accounting for 8.92% of the total. Among the collected high-protein peanut germplasms, 15 varieties were classified as high-oil and high-protein, 14 as high-protein and high-oleic acid, and 2 as high-oil, high-protein, and high-oleic acid, namely “Heizhenzhu 2” and “Pukehua 10” (Table 2). These findings suggest that high-protein peanut varieties combining superior yield with multiple quality traits remain scarce. Breeding such multi-trait varieties, particularly those high in oil, protein, and oleic acid, requires advanced biotechnological approaches. Nevertheless, China has already developed several high-quality varieties and is expected to produce more in the future.

**Figure 2:**
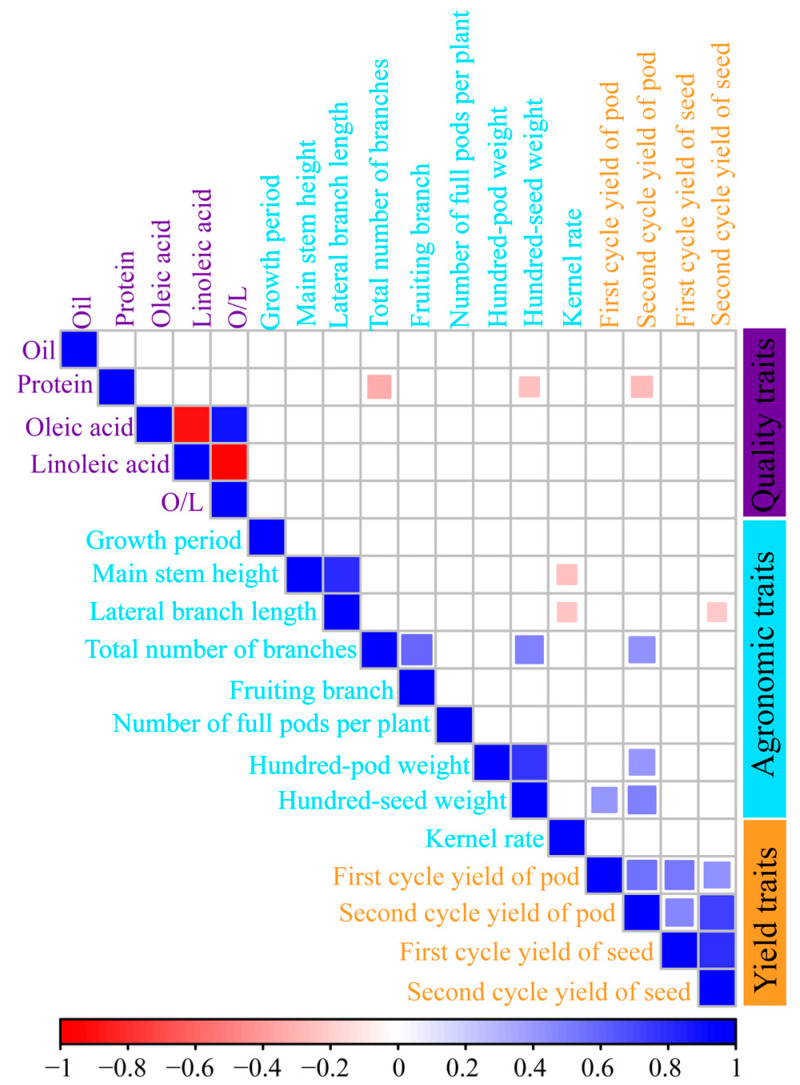
Correlation analysis among quality traits, agronomic traits, and yield traits of high-protein peanuts in China. It was conducted using R4.4.0 and R Studio (R 4.5.0), with the “corrplot” package used to analyze the significance of trait correlations. Blue squares indicate positive correlations and red squares indicate negative correlations.

Analysis of 17 agronomic, quality, and yield traits across the 168 high-protein peanut germplasms revealed a significant negative correlation between protein and oil content. This trade-off suggests that increasing protein content often results in reduced oil accumulation, posing a challenge for breeding varieties high in both traits. High yield and quality in peanut varieties are the result of multiple traits working together, requiring breeders to use this correlation to identify key genes controlling oil and protein synthesis through gene editing or hybridization, and to optimize seed composition through gene regulation to breed varieties with high oil content, high protein content, and high oleic acid content (Figure 2). This indicates that under conventional breeding, an increase in protein content typically leads to a decrease in oil content due to their competitive metabolic pathways [16]. For instance, *GmMFT* simultaneously regulates the oil and protein content as well as seed size in soybeans. Knockout plants showed a significant decrease in seed oil content and a significant increase in protein content [17]. However, recent studies suggest that this trade-off can be mitigated by pyramiding favorable alleles through advanced techniques such as marker-assisted selection or gene editing. The strategy of simultaneous improvement of protein and oil content has been proposed for peanut [7]. Therefore, breeders may leverage these negative correlations to identify key genes controlling oil and protein synthesis, e.g., *AhFAX1* and *DGAT1* [12] and optimize seed composition through targeted gene regulation, enabling the development of varieties with combined high protein, high oil, and high oleic acid content [18,19].

In addition, there was a significant positive correlation between main stem height and lateral branch length, and both were significantly negatively correlated with kernel yield, indicating that excessive main stem height leads to larger plant size, which results in vigorous vegetative growth and excessive nutrient consumption, thereby affecting kernel yield. Therefore, in agricultural production, the size of peanut plants should be appropriately controlled, highlighting the importance of controlling plant architecture in cultivation. The number of fruiting branches was significantly positively correlated with the total number of branches and the number of full pods per plant, indicating that an increase in the total number of branches directly affects the number of fruiting branches and indirectly influences the number of full pods per plant. Therefore, moderate increases in branch number may be beneficial. The growth period was significantly positively correlated with the total number of branches, 100-pod weight, and 100-kernel weight, indicating that a longer growth period allows for more nutrient absorption, which better contributes to an increase in the total number of branches and, consequently, peanut yield. Protein content was significantly positively correlated with 100-pod weight, indicating that quality traits and yield traits influence each other, suggesting that the breeding of high-yield, high-quality peanut varieties is feasible. Protein content was significantly negatively correlated with the total number of branches, while the number of fruiting branches was significantly positively correlated with 100-pod weight. The total number of branches and growth period were significantly positively correlated with 100-pod weight and 100-kernel weight, indicating that agronomic traits, yield traits, and quality traits influence each other. Therefore, the relationships between traits can be used to infer invisible quality traits and predict yield, thereby improving the efficiency of breeding high-yield, high-quality peanut varieties. It should be noted that the correlation analysis presented here is based on mean trait values across multiple environments without statistical adjustment for environmental effects. Therefore, these correlations are descriptive and may include both genetic and environmental components. Future studies incorporating mixed models or best linear unbiased predictions would help to better elucidate the genetic relationships among these traits.

### 2.3. Disease Resistance and Environmental Adaptability

Disease and pest outbreaks are major factors that suppress yield [20], and high levels of resistance to many important biotic and abiotic stresses are not available in the cultivated gene pool [21]. Enhancing their disease resistance can not only improve the stability and adaptability of peanuts but also contribute to green agriculture and sustainable development. The trait integration based on breeding goals to cultivate disease-resistant and high-protein peanut varieties can not only prevent peanut diseases but also increase the yield and economic benefits of high-protein peanuts. The data on disease resistance for the 168 high-protein peanut varieties summarized here were systematically collected from the official Variety Registration Inquiry System of the Seed Management Division of the Ministry of Agriculture and Rural Affairs, P.R. China and the Peanut Database. These platforms provide standardized and validated data on disease resistance from multi-location regional trials, which are mandatory for national variety registration in China. The resistance levels to various diseases (e.g., bacterial wilt, leaf spot, rust) for each variety are evaluated according to the national uniform technical standards for crop variety testing (NY/T 2401-2013) [22]. This ensures the consistency and comparability of the resistance data across different varieties and sources.

**Figure 3:**
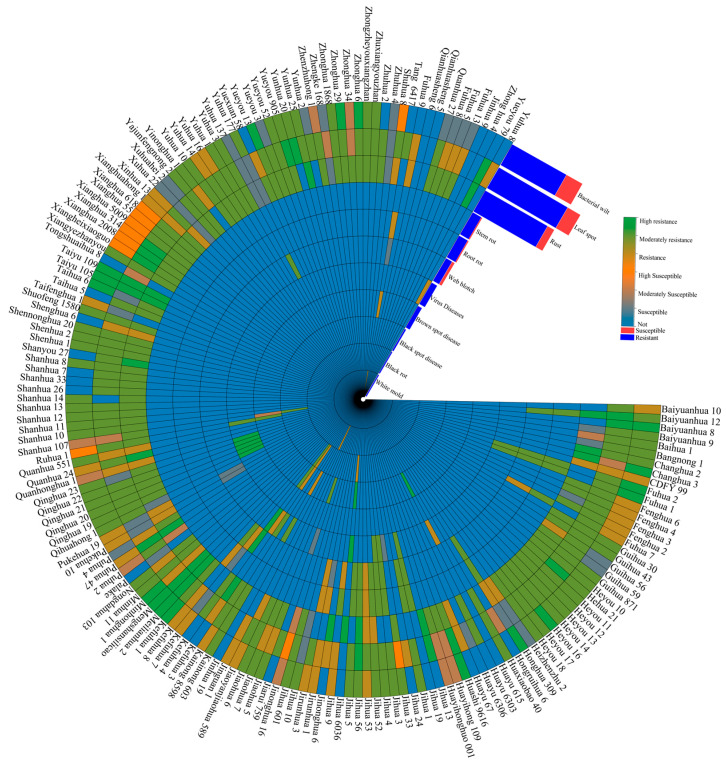
Comprehensive analysis of disease resistance in high-protein peanut in China. Disease resistance phenotyping employed TBtools (v2.337) bioinformatics suite to create multidimensional thermodynamic circular heatmaps for eleven pathogen resistance categories, with chromatographic gradient encoding techniques delineating resistance levels.

The disease resistance profiles of high-protein peanut varieties in China have been evaluated for major diseases including bacterial wilt, leaf spot, rust, stem rot, root rot, web blotch, virus diseases, brown spot disease, black spot disease, black rot, white mold were investigated (Figure 3). Among them, the variety “Kefuhua 4” exhibits resistant (including resistance, moderate resistance, and high resistance) to six diseases and is suitable for cultivation in Jilin Province. Nine high-protein peanut varieties are resistant to five diseases, including “Baiyuanhua 12”, “Fuhua 2”, “Fuhua 1”, “Jihua 3”, “Jihua 4”, “Jihua 52”, “Jihua 53”, “Jihua 56”, and “Kefuhua 3”. All are adapted to Jilin Province, with “Fuhua 2” and “Fuhua 1” also suitable for Heilongjiang, Inner Mongolia, Xinjiang, and Jiangsu. Seventeen high-protein peanut varieties show resistance to four diseases; 65 to three diseases; 55 are resistant to two diseases; nine are resistant to one disease; eight high-protein peanut varieties show no resistance to tested diseases, including “Hongruihua 6”, “Huayu 6306”, “Jinonghua 16”, “Palake”, “Tongshuaihua 8”, “Xuhua 22”, “Zhengke 168”, “Zhuhua 4”; and four varieties have not undergone disease resistance evaluation, including “Qianhuasheng 6”, “Qianhuasheng 5”, “Jinhau 9”, “Zhonghua 4”(Figure 3). These results indicate that while most high-protein peanut varieties in China confer resistance to two to four diseases, few exhibit multi-disease resistance. Some varieties lack documented resistance or remain insufficiently tested. Analysis of disease resistance types revealed that 105 high-protein peanut varieties are resistant to bacterial wilt, while 31 are susceptible (including susceptible, moderately susceptible, and highly susceptible); 137 are resistant to leaf spot, while 22 are susceptible; and 121 are resistant to rust, while 13 are susceptible. Resistance to other diseases, such as white mold, root rot, pod rot, viral diseases, and brown spot, has been less extensively studied. Overall, high-protein peanut varieties in China still lack comprehensive disease resistance.

Wild peanut resources have rich genetic diversity and are an important source for disease resistance breeding [23]. To enrich the genetic background of cultivated peanut varieties, a fundamental strategy involves the introduction of valuable traits from wild species, particularly those contributing to high-protein content and multi-disease resistance. Wild peanut relatives serve as excellent sources of resistance genes [23], and have been successfully utilized to introduce broad-spectrum resistance against major pathogens, including leaf spots [24] and thrips-transmitted tomato spotted wilt orthotospovirus [25]. Both protein content and disease resistance in peanuts are quantitative traits controlled by multiple genes, making it difficult to achieve through conventional hybridization. Several molecular markers related to peanut protein and disease resistance have been developed and applied in breeding practices. For example, bacterial wilt, caused by Ralstonia solanacearum, is another devastating disease affecting peanuts, with about 350 hosts. QTL-seq analysis of the Yuanza 9102 (resistant) × Xuzhou 68-4 (susceptible) RIL population identified a 2.07 Mb genomic region containing several genes related to bacterial wilt resistance [26,27]. The data compilation of the disease resistance of 168 high-protein peanut varieties in China revealed that their disease resistance is still not comprehensive. Next, efforts should focus on breeding high-protein varieties with broad-spectrum resistance and elucidating the mechanisms underlying peanut disease resistance. Such advances will provide a theoretical basis for disease management and support a sustainable future for the peanut industry.

### 2.4. Pedigree Analysis of High-Protein Peanut Varieties

**Figure 4:**
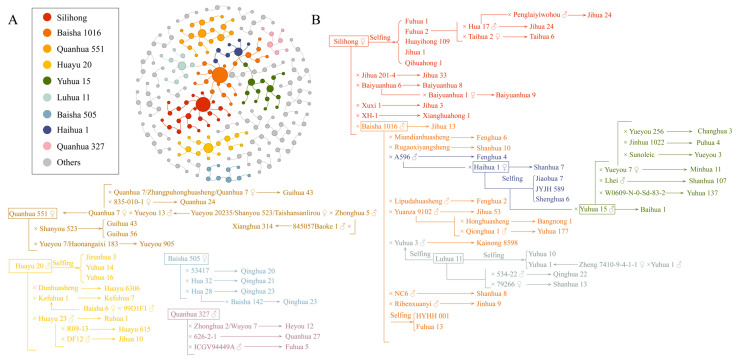
Pedigree analysis of high-protein peanut in China. (**A**): Total pedigree map. (**B**): Sub-pedigree map of the important parental pedigree relationship. Pedigree network construction was implemented through Gephi 0.10.1 graph theory platform, applying force-directed algorithms to optimize topological structure based on genealogical data from 168 high-protein peanut varieties and their parental lines. ♂ represents the male parent, and ♀ represents the female parent. The unmarked part and the boxed part were crossed, and the boxed part was the paternal part.

Pedigree analysis serves as a fundamental methodology in plant breeding, offering critical insights into the genetic inheritance of key traits and guiding parental selection. Through systematic pedigree examination, the genetic diversity within peanut germplasm resources can be elucidated, enabling the identification of parental lines with superior characteristics. This approach provides a scientific basis for targeted breeding of high-protein traits and significantly enhances the efficiency of peanut variety development. This review visualized the genetic relationships among 168 high-protein peanut varieties and their parental lines (Figure 4A) and analyzed key pedigree details (Figure 4B). Among the core parental varieties, “Baisha 1016” contributed to the development of 12 high-protein varieties (7.1%), while “Silihong” produced 11 high-protein varieties (6.5%). In hybridization breeding, “Baisha 1016” exhibited the highest frequency of direct or indirect utilization frequency, followed by “Silihong”, “Huayu 20”, “Luhua 11”, “Haihua 1”, “Yueyou 7”, “Quanhua 551”, and “Baisha 505”. From a genetic breeding perspective, the development of high-protein peanut varieties predominantly relies on interparental hybridization, complemented by parental selfing and selection of mutant lines through mutagenesis. Among the 168 varieties, 127 (75.6%) were developed through crossbreeding, while 35 (20.8%) originated from self-pollination strategies. Parental lineage information was incomplete for six cultivars, constituting 3.6% of the germplasm collection. These findings collectively suggest that the genetic foundation of high-protein peanut breeding remains narrow, necessitating the introduction of exotic germplasm or innovative hybridization strategies to enhance genetic diversity.

The pedigree map of 168 high-protein peanut varieties in China are derived from a limited number of cultivated varieties, often through selection within self-pollinated progeny. It is worth noting that wild peanut germplasm and local varieties contain rich genetic resources for high protein, disease resistance, and drought tolerance. Therefore, future breeding efforts should prioritize the integration of these diverse genetic resources. Combining conventional breeding techniques with modern molecular approaches will facilitate the pyramiding of high-protein traits with genes conferring disease resistance and other agronomic advantages, ultimately leading to the development of breakthrough varieties with high quality, high yield, and enhanced resilience.

### 2.5. Identification of Protein-Related QTLs

QTL refers to gene regions that control quantitative traits such as yield, quality, and disease resistance. These traits are typically controlled by multiple genes, and the effects of these genes are cumulative, hence they are referred to as polygenic traits [28]. The goal of QTL research is to locate these gene regions controlling quantitative traits through genetic markers (such as Simple Sequence Repeats, single nucleotide polymorphism (SNP), etc.), thereby providing a foundation for molecular marker-assisted selection and gene editing in breeding technologies [29]. Researchers have employed various genetic populations, including recombinant inbred lines and F_2_ populations, to conduct QTL mapping for protein content under multiple environments. These efforts have led to the identification of several protein-related QTLs [30,31]. Additionally, GWAS using diverse peanut germplasms in different environments have successfully developed molecular markers for genetic loci related to yield and quality traits. In reported studies, a mapping population derived from the TMV2 × TMV2-NLM cross comprising 432 RILs revealed seven protein content-related QTLs located on chromosomes 5, 10, and 16 [32]. Similarly, a hybrid population from TG 26 × GPBD 4 including 146 inbred lines identified several QTLs for protein content, explaining phenotypic variation ranging from 21.12% to 37.51% [30]. In another study, a population of 329 RILs from Yuhua 15 × W 1202 was used to construct a high-density genetic map with 213,868 SNP markers, leading to the detection of 29 protein-related QTLs, accounting for 18.2% to 29% of phenotypic variation [14]. Using QTL-seq, a major QTL named qSPCB10.1 was identified within the 117.14–133.2 Mb interval on chromosome B10 [7]. Guo et al. performed GWAS on 199 peanut accessions using a high-density map with 631,988 SNPs and detected two significant loci associated with protein content [12]. Zhang et al. conducted a GWAS on 120 U.S. peanut genotypes with 13,382 SNPs and identified 22 QTLs related to seed protein content, among which AX-147219970 on chromosome A04 was a prominent multi-trait locus influencing seed composition [33].

**Figure 5:**
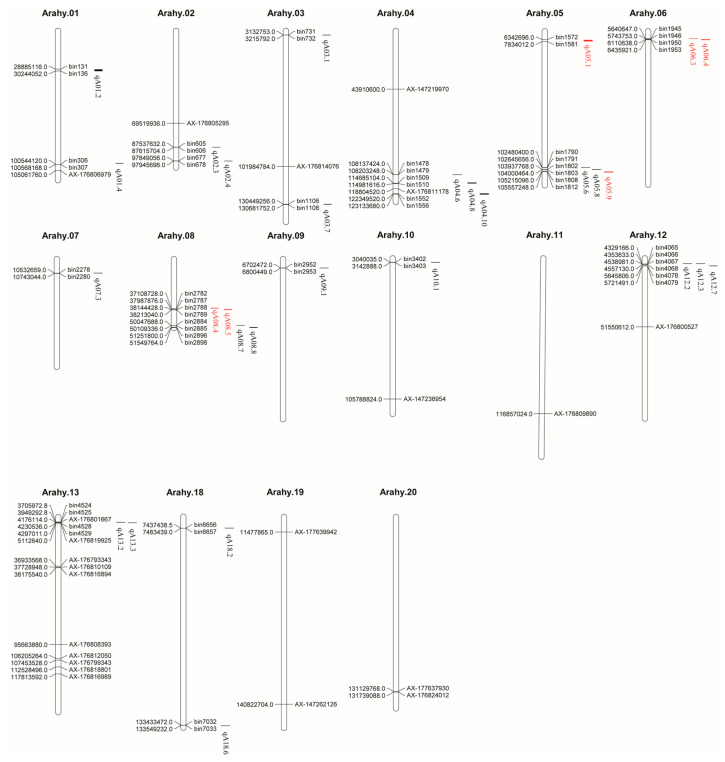
Physical map of QTLs associated with protein content in peanut. Note: Red marks are associated loci with phenotypic variation explained greater than or equal to 10%.

These studies provide valuable insights into the genetic architecture of peanut protein content and supply potential molecular tools for quality improvement, thereby supporting the efficient breeding of high-protein varieties. However, it is important to note that most reported QTLs are population-specific, which limits their broader applicability and complicates marker validation and deployment. Integrating mapping results from independent studies into a unified physical map is essential to identify consensus molecular markers for breeding. By consolidating information from QTL mapping and association studies related to protein and oil content, and aligning marker positions with the peanut tetraploid reference genome, a physical map of genetic loci associated with protein content has been constructed. This map comprises 106 loci in total; however, 26 could not be accurately mapped due to missing marker sequences (14 loci) or the absence of corresponding physical position information (12 loci). Among the 43 protein-related QTLs, 14 remain unmapped owing to unresolved chromosomal locations (Figure 5). In summary, the number of reliably mapped QTLs and genetic loci controlling peanut protein content remains limited, and their underlying regulatory mechanisms are not yet fully understood. These gaps significantly constrain the breeding of high-protein peanut varieties. Therefore, further identification and functional characterization of genetic loci regulating seed protein content are critically needed to accelerate breeding progress.

### 2.6. Candidate Genes of Protein Content in Peanut Seeds

The accumulation of protein in peanut kernels is regulated by complicated mechanisms, yet the underlying regulatory networks remain incompletely elucidated. Recent studies have identified *AhFAX1* as a key candidate gene governing seed protein content. *AhFAX1*, implicated in fatty acid and lipid homeostasis, functions as a putative plastid-localized fatty acid transporter. Transgenic validation in *Arabidopsis* demonstrated that overexpression of *AhFAX1* significantly enhances seed oil and protein content while increasing seed size and weight [34]. GWAS and transcriptomic analyses further pinpointed *Ahy_A09g041582* (a homolog of *LAC15*), located on chromosome A09, as a critical regulator of plant growth, development, and amino acid accumulation in seeds. Notably, it exhibits elevated expression in peanut pericarps and seeds, suggesting its role in modulating amino acid biosynthesis [35]. Integrated multi-omics approaches, including broad-target metabolomics, quantitative lipidomics, and transcriptomics, have uncovered three co-expressed modules harboring pivotal genes controlling lipid and protein content in peanut kernels [16].

## 3. Challenges and Future Directions

The genus Arachis contains approximately 80 annual and perennial species. Based on morphological similarity, cross-compatibility, and viability of hybrids, the genus is divided into nine taxonomic sections. The Arachis section consists of the largest number of species, with 2n = 2x = 20 or 2n = 4x = 40. The cultivated A. hypogaea (2n = 4x = 40) belongs to the Arachis section. A. hypogaea is further divided into two subspecies, Fastigiata Waldron and hypogaea Krap. et Rig., with four botanical varieties, Var. Vulgaris, Var. Fastigiata, Var. Peruviana, and Var. aequatoriana, and two cultivars, Var. Hypogaea and Var. hirsuta [36]. These extensive germplasm resources provide a solid foundation for peanut breeding. High-protein peanut breeding is an important research direction in agricultural science. Peanut protein contains all 20 amino acids and is the richest source of arginine (12.5% of total protein) [6], which is associated with multiple health benefits such as cardiovascular disease prevention, weight management, and satiety [37]. The aggregation and improvement of quality and agronomic traits in high-protein peanut varieties will give them a market advantage over conventional protein content varieties. However, combining this trait with other desirable traits such as high yield, high oleic acid, and high oil content can be challenging. A significant negative correlation between oil content and protein content, oleic acid content and linoleic acid content, and linoleic acid content and the oil/linoleic acid ratio [18]. Therefore, under the premise of high protein content, breeding new high-protein, high-oil, high-oleic acid, and high-yield peanut varieties could better meet the diverse needs of both markets and processing enterprises for high-protein peanuts. Traditional breeding methods can no longer meet this demand, and advanced breeding techniques such as molecular breeding, radiation mutagenesis, and high-protein gene introgression are needed. Currently, there are relatively few reports on this topic.

**Figure 6:**
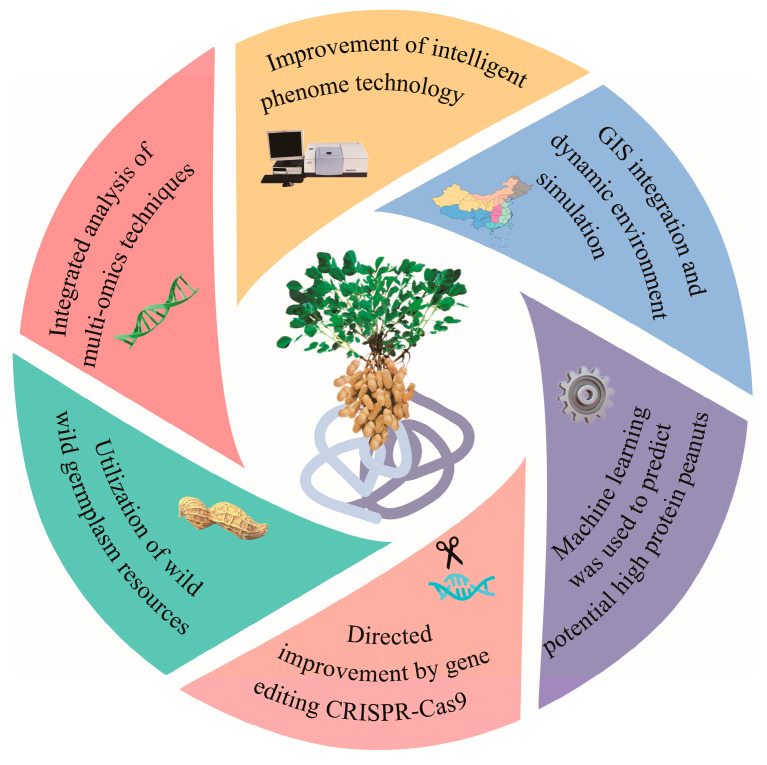
Future perspective on breeding of high-protein peanuts.

High-protein peanut breeding in China still relies mainly on traditional breeding methods, but with the rapid development of molecular breeding technologies, the application of genomics, molecular marker-assisted selection, and other modern techniques will significantly improve breeding efficiency. In terms of breeding strategies, market demand should be the guide, focusing on breeding new varieties with comprehensive traits such as high protein, high oleic acid, strong disease resistance, and excellent stress resistance. Future research should focus on integrating genetics, molecular biology, and metabolomics to conduct dynamic analysis and reveal the formation mechanisms of high-protein traits in peanuts (Figure 6). At the same time, intelligent phenotyping technologies (such as non-destructive phenotyping and AI-based imaging) should be used for precise screening, and gene-environment interaction patterns should be analyzed to establish predictive models for directional optimization of stress-resistant germplasm. In addition, wild germplasm resources should be fully utilized, and genetic limitations should be overcome using cutting-edge technologies such as gene editing, ultimately building an efficient and sustainable high-protein breeding system, providing an innovative paradigm for the development of functional crops and the intensive use of agricultural resources. Furthermore, future breeding programs should prioritize enhancing resistance to currently less-studied but economically significant diseases, such as white mold, root rot, and viral diseases. An integrated strategy combining standardized field phenotyping in multi-environment trials, high-throughput molecular screening (e.g., GWAS, QTL mapping), and the exploitation of wild relatives’ genetic diversity is recommended. Advanced techniques like genomic selection and gene editing should be employed to pyramid resistance genes into high-protein elite varieties, thereby achieving durable and broad-spectrum disease resistance.

## Figures and Tables

**Table 1:** Variation analysis of main traits of 168 high-protein peanut varieties.

Traits	Mean	Range	SD	CV%
Oil content (%)	50.90	36.40–59.32	2.92	5.75
Protein (%)	29.52	27.99–36.40	1.44	4.86
Oleic acid (%)	47.02	33.00–90.60	10.64	22.63
Linoleic acid (%)	32.56	1.92–44.72	8.97	27.55
Growth period (d)	122.25	100.00–138.00	7	5.78
Main stem height (cm)	43.79	16.87–67.60	8.89	20.30
Lateral branch length (cm)	46.74	8.50–75.20	9.60	20.54
Total number of branches	7.95	3.66–20.00	2.09	26.29
Fruiting branch	6.74	3.51–14.48	1.48	22.00
Number of full pods per plant	16.96	6.70–50.00	5.16	30.40
Hundred-pod weight (g)	191.18	69.90–332.00	40.52	21.20
Hundred-seed weight (g)	76.25	32.23–169.70	17.70	23.22
Kernel rate (%)	70.86	61.66–85.00	3.38	4.78
First cycle yield of pod (kg)	300.55	110.10–520.60	69.33	23.07
Second cycle yield of pod (kg)	286.81	130.29–511.00	69.40	24.20
First cycle yield of seed (kg)	230.58	115.51–505.80	59.29	25.72
Second cycle yield of seed (kg)	217.44	128.71–388.40	52.26	24.03

**Table 2:** High-protein peanut varieties with elevated oil and/or oleic acid content in China.

Variety Name	Protein (%)	Oil (%)	Oleic Acid (%)
Heizhenzhu 2	28.90	55.91	79.24
Pukehua 10	28.09	55.40	76.07
Yuhua 14	28.11	59.32	41.5
Yuhua 16	28.20	58.94	40.7
Heyou 10	28.33	56.83	45.2
Zhuxiangyouzhan	28.65	56.13	46.2
Ruhua 1	29.10	56.00	51.7
Hehua 21	28.90	55.87	48.4
Zhongzheyouxiangzhan	28.35	55.62	45.3
Yueyou 3	29.40	55.49	45.4
CDFY 99	28.43	55.46	45.6
Shanhua 11	28.22	55.46	51.5
Xiangyezhanyou	28.62	55.35	46.1

## Data Availability

No new data were created or analyzed.

## Supplementary Materials

The following supporting information can be downloaded at: https://www.mdpi.com/article/10.3390/plants14182917/s1, Table S1: List of different high oleic cultivars released in China.
